# Temporal changes in clinical and radiographic variables in dogs with preclinical myxomatous mitral valve disease: The EPIC study

**DOI:** 10.1111/jvim.15753

**Published:** 2020-03-22

**Authors:** Adrian Boswood, Sonya G. Gordon, Jens Häggström, Martin Vanselow, Gerhard Wess, Rebecca L. Stepien, Mark A. Oyama, Bruce W. Keene, John Bonagura, Kristin A. MacDonald, Mark Patteson, Sarah Smith, Philip R. Fox, Karen Sanderson, Richard Woolley, Viktor Szatmári, Pierre Menaut, Whitney M. Church, M. Lynne O'Sullivan, Jean‐Philippe Jaudon, Jan‐Gerd Kresken, John Rush, Kirstie A. Barrett, Steven L. Rosenthal, Ashley B. Saunders, Ingrid Ljungvall, Michael Deinert, Eric Bomassi, Amara H. Estrada, Maria J. Fernandez Del Palacio, N. Sydney Moise, Jonathan A. Abbott, Yoko Fujii, Alan Spier, Michael W. Luethy, Roberto A. Santilli, Masami Uechi, Anna Tidholm, Christoph Schummer, Philip Watson

**Affiliations:** ^1^ Department of Clinical Science and Services, The Royal Veterinary College Hatfield, Hertfordshire UK; ^2^ Department of Small Animal Clinical Sciences, Texas A&M University College Station Texas; ^3^ Department of Clinical Sciences Swedish University of Agricultural Sciences Uppsala Sweden; ^4^ Biometrie and Statistik, Hannover Germany; ^5^ Clinic of Small Animal Medicine University of Munich Munich Germany; ^6^ School of Veterinary Medicine, Medical Sciences University of Wisconsin Madison Madison Wisconsin; ^7^ Clinical Studies‐Philadelphia, MJR‐VHUP‐Cardiology University of Pennsylvania Philadelphia Pennsylvania; ^8^ Department of Clinical Sciences North Carolina State University, College of Veterinary Medicine Raleigh North Carolina; ^9^ Department of Veterinary Clinical Sciences The Ohio State University Columbus Ohio; ^10^ Animal Care Center Rohnert Park California; ^11^ HeartVets @ Vale Referrals, The Animal Hospital Stinchcombe, Dursley Glos UK; ^12^ Sarah Smith Cardiology Derby UK; ^13^ Department of Cardiology and Caspary Research Institute, Animal Medical Center New York New York; ^14^ Rocky Mountain Veterinary Cardiology Boulder Colorado; ^15^ Cardio Respiratory Pet Referrals Mordialloc, Melbourne Victoria Australia; ^16^ Faculty of Veterinary Medicine, Clinical Sciences of Companion Animals Utrecht University Utrecht The Netherlands; ^17^ Clinique Vétérinaire AQUIVET Eysines France; ^18^ Desert Veterinary Medical Specialists Phoenix Arizona; ^19^ Department of Companion Animals Atlantic Veterinary College, University of Prince Edward Island Charlottetown Prince Edward Island Canada; ^20^ Clinique Veterinaire Des Etangs Villars Les Dombes France; ^21^ Clinic for Small Animals Kaiserberg Duisburg Germany; ^22^ School of Veterinary Medicine, Clinical Sciences Tufts University Cummings North Grafton Massachusetts; ^23^ Cardiology VCA West Los Angeles Los Angeles California; ^24^ CVCA Cardiac Care for Pets Towson Maryland; ^25^ Fachtierarztpraxis Am Sandpfad Wiesloch Germany; ^26^ Centre Hospitalier Vétérinaire des Cordeliers, Cardiology, Meaux Paris France; ^27^ University of Florida College of Veterinary Medicine, Small Animal Clinical Sciences Gainesville Florida; ^28^ Medicina y Cirugía Animal Universidad de Murcia Murcia Spain; ^29^ Clinical Sciences, College of Veterinary Medicine Cornell University Ithaca New York; ^30^ Department of Small Animal Clinical Sciences Virginia‐Maryland College of Veterinary Medicine, Virginia Tech Blacksburg Virginia; ^31^ Azabu University, Surgery 1 Kanagawa Japan; ^32^ Blue Pearl Veterinary Partners Tampa Florida; ^33^ Chicago Veterinary Emergency and Specialty Center Chicago Illinois; ^34^ Cardiology Clinica Veterinaria Malpensa Varese Italy; ^35^ Japan Animal Specialty Medical Institute Inc., JASMINE Veterinary Cardiovascular Medical Center Yokohama Japan; ^36^ Djursjukhuset Albano Stockholm Sweden; ^37^ Boehringer Ingelheim, Animal Health Ingelheim am Rhein Germany

**Keywords:** canine, cardiovascular, clinical epidemiology, clinical trials, heart failure, physical examination, radiology and diagnostic imaging

## Abstract

**Background:**

The Evaluation of pimobendan in dogs with cardiomegaly caused by preclinical myxomatous mitral valve disease (EPIC) study monitored dogs with myxomatous mitral valve disease (MMVD) as they developed congestive heart failure (CHF).

**Objectives:**

To describe the changes in clinical and radiographic variables occurring as dogs with MMVD and cardiomegaly develop CHF, compared to similar dogs that do not develop CHF.

**Animals:**

One hundred and thirty‐five, and 73 dogs that did or did not develop CHF, respectively.

**Materials and methods:**

The following variables were evaluated in 2 groups of dogs (dogs that did or did not develop CHF): Heart rate (HR), clinic respiratory rate (RR), home‐measured resting respiratory rate (RRR), rectal temperature (RT), body weight (BW), and vertebral heart sum (VHS). Absolute value and rate of change of each variable were calculated for each day a dog was in study. Daily means were calculated and plotted against time. The onset of CHF or last visit before leaving the study were set as reference time points.

**Results:**

The most extreme values and rate of change occurred in variables immediately before onset of CHF. Vertebral heart sum increased earliest. Heart rate, RR, and RRR also increased. Rectal temperature and BW decreased. Increases in RR and RRR were most extreme and occurred immediately before CHF.

**Conclusions and Clinical Importance:**

Dogs with MMVD and cardiomegaly experience increases in HR, RR, RRR, and VHS, and decreases in BW and RT as they develop CHF. The variables with highest absolute change and rate of change were RR and RRR. These findings reinforce the value of RR and RRR as indicators of impending or incipient CHF.

**Abbreviations**
BPM: beats per minute
BW: body weight
CHF: congestive heart failure
EPIC: Evaluation of pimobendan in dogs with cardiomegaly caused by preclinical myxomatous mitral valve disease
HR: heart rate
IQR: inter quartile range
LA/Ao: left atrial‐to‐aortic root ratio
LVIDDN: left ventricular internal diameter in diastole‐normalized
MMVD: myxomatous mitral valve disease
MR: mitral regurgitation
RR: respiratory rate (in clinic)
RRR: resting respiratory rate (owner measured)
RT: rectal temperature
VHS: vertebral heart sum

## 1 INTRODUCTION

The “Evaluation of pimobendan in dogs with cardiomegaly caused by preclinical myxomatous mitral valve disease” (EPIC) study was a multicenter, blinded, randomized, placebo‐controlled clinical trial evaluating the effect of pimobendan in delaying the onset of clinical signs in dogs with cardiac enlargement secondary to myxomatous mitral valve disease (MMVD). The main findings and longitudinal results have been published previously.^1^, ^2^ Longitudinal analysis indicated that a number of clinical, laboratory, radiographic, and echocardiographic variables changed significantly as dogs developed heart failure.^1^ Dogs receiving pimobendan were indistinguishable from those receiving placebo at the time of the onset of congestive heart failure (CHF).^1^

Previous studies have identified not only the absolute changes in clinical, radiographic, and echocardiographic variables in patients with progressive MMVD, but the time course over which these changes occur. One study documented the change in vertebral heart sum (VHS) that occurred in Cavalier King Charles Spaniels before the onset of CHF.^3^ The change in heart size occurred over approximately 1 year and was maximal immediately before the onset of CHF.^4^ Longitudinal changes in echocardiographic indicators of heart size,^5^ heart rate (HR), and heart rate variability^6^ before patients succumb to MMVD indicate a similar pattern with an increasing rate of change of the monitored variables as the disease progresses. The increase in heart size before the onset of CHF occurs so consistently that heart size has been validated as a component of a regression equation that can be used with reasonable accuracy to predict the onset of CHF.^7^ Dogs developing CHF in the EPIC study showed increased heart size and HR at the time of onset of CHF compared to measurements made at baseline.^1^

Respiratory rate (RR), measured in the clinic, and client‐measured resting respiratory rate (RRR), measured in the dog's home environment, have been shown to be higher in dogs in CHF^8^, ^9^ returning to more normal values once signs of heart failure are medically controlled.^10^ The longitudinal change in RR and RRR in a large population of dogs as they develop CHF has not been described previously. Dogs with preclinical left‐sided heart disease have been shown to have a RRR that usually is <25 breaths per minute.^11^ Respiratory rate measured in the clinic was significantly higher at the time of onset of CHF in dogs in the EPIC study compared to results obtained at baseline. The RRR measured by owners in the home environment was not available at the baseline visit for comparison with results obtained immediately before CHF.^1^

As well as the previously reported changes in HR, heart size, and RR, dogs in the EPIC study that developed CHF experienced decreases in rectal temperature and body weight as they developed CHF.^1^

In the EPIC study, over 100 dogs were monitored as they developed CHF.^2^ Data from this population therefore provide a unique opportunity to determine not only the magnitude of the change in clinical and radiographic variables as CHF develops, but the time course over which those changes occur.

The aim of our current study is to describe, in a group of dogs with stage B2^12^ MMVD, the temporal changes in clinical and radiographic variables that occur before development of CHF and to contrast the changes in dogs that developed CHF to a population of dogs known not to have developed CHF over the duration of the study.

## 2 MATERIALS AND METHODS

### 2.1 Trial design

The EPIC trial was a prospective multicenter, blinded, randomized, placebo‐controlled study. Complete and detailed description of the study and longitudinal changes in measured variables have been published.^1^, ^2^ The study was approved by an ethical review committee at each site where this was required.

### 2.2 Dogs

#### 2.2.1 Enrollment criteria

Dogs were eligible for participation in the study provided the owner had given informed consent. To be eligible for inclusion, a dog had to be ≥6 years of age, have a body weight (BW) ≥4.1 and ≤15 kg, have a characteristic systolic heart murmur of moderate‐to‐high intensity (grade ≥ 3/6) with maximal intensity over the mitral area, have echocardiographic evidence of advanced MMVD defined as characteristic valvular lesions of the mitral valve apparatus, mitral regurgitation (MR) on the color Doppler echocardiogram, and have echocardiographic and radiographic evidence of cardiomegaly defined as a left atrial‐to‐aortic root ratio (LA/Ao) ≥1.6 measured in a short‐axis view,^13^ BW‐normalized left ventricular internal diameter in diastole (LVIDDN)^14^ ≥1.7 and a VHS >10.5.^15^

#### 2.2.2 Exclusion criteria

Dogs were excluded from the study if they had any of the following: known clinically important systemic or other organ‐related disease that was expected to limit the dog's life expectancy or required chronic administration of cardiovascular medication precluded as part of the trial. Dogs with hypothyroidism could be included provided the investigator deemed them clinically stable on treatment. Dogs with current or previous evidence of cardiogenic pulmonary edema, pulmonary venous congestion or both, cardiac disease other than MMVD, clinically significant supraventricular, ventricular tachyarrhythmias or both (ie, requiring antiarrhythmic treatment), or evidence of pulmonary hypertension considered to be clinically relevant (right ventricular: right atrial pressure gradient >65 mmHg) were excluded. Dogs with a history of chronic or recent administration (>14 days of duration or within 30 days of intended enrollment) of any precluded medication were excluded. In the event that, before study enrollment, a dog had received short‐term treatment (<14 days) with a precluded agent, but was no longer receiving treatment and had not received it within 30 days of intended enrollment, then the dog was eligible for inclusion. Dogs that were pregnant or lactating were not eligible for enrollment.

Details of study sites, randomization and blinding, trial medication, concomitant treatment, and data management have been described previously.^2^

#### 2.2.3 Schedule of events

Before inclusion, a case history was taken for each dog. At a baseline visit, dogs underwent physical examination, echocardiography, thoracic radiography, and routine hematology and blood biochemistry. Reexaminations were scheduled at 35 days after the baseline visit, approximately 4 months after baseline and every 4 months thereafter. Details of examinations that were undertaken on each visit are provided in Table 1.

**Table 1:** Schedule of procedures undergone by animals remaining in the per‐protocol population of the study at different examinations

	Baseline visit	Baseline +35 days (±7 days)	Baseline +4 months^a^	Baseline +8 months^b^	Event
Physical examination	X	X	X	X	X
Thoracic radiograph	X			X	X
Owner measured respiratory rate		X	X	X	X

a Four months from baseline visit and every 8 months thereafter.

b Eight months from baseline visit and every 8 months thereafter.

#### 2.2.4 Clinical evaluation

At inclusion, dog characteristics such as breed, age, sex, and neuter status were noted. The BW, HR, RR, and rectal temperature (RT) were measured at each visit.

#### 2.2.5 Thoracic radiography

Thoracic radiography was performed at the baseline visit, 8 months after baseline and every 8 months thereafter for as long as the dog remained in the per‐protocol population. It also was performed at the time a dog was considered to have developed signs of CHF. Right lateral and dorsoventral projections were used to evaluate the thorax. Cardiac size was assessed by the VHS method,^15^ and pulmonary edema and congestion were recorded, when considered to be present, by the attending cardiologist.

#### 2.2.6 Resting respiratory rate

Owners also were asked to measure RRR before every reexamination. Instruction was provided to owners who were advised to count the respiratory rate over 1 minute, in the dog's home environment, within the week before each reexamination. Ideally, determination was performed on several days and the average of the measurements obtained was recorded as a single value at the corresponding visit.

#### 2.2.7 Primary endpoint

The primary endpoint was a composite of the development of left‐sided CHF verified by an endpoint committee,^2^ euthanasia for a cardiac reason, or death presumed to be cardiac in origin.

### 2.3 Statistical methods

For those dogs with verified CHF (Group CHF), the day of confirmed CHF was considered day 0. The day of measurement of different variables was expressed as the number of days before day 0. For example, if a measurement was taken 7 days before the onset of CHF, this day would be considered day −7.

For comparison, a group of dogs that were known not to have developed CHF (Group no‐CHF) for at least 4 months after their final examination were included. This group consisted of dogs that were censored at the time of study closure that were known not to have experienced CHF by that time (March 1, 2015), and for which sufficient data were available to contribute to the analyses. Day 0 for no‐CHF dogs was defined as the day of the last visit on which all radiographic and clinical examination data were obtained (see Table 1) that was at least 4 months before closure of the study.

The values for BW were indexed to the baseline value of this variable because of the large range of values seen in the population. Indexing was performed according to the formula 100 × (observed BW [kg])/(BW baseline [kg]). Therefore, dogs that experienced weight loss would have values less than 100.

Observations were evaluated for plausibility. All respiratory rate (RR and RRR) observations ≥100/min were assumed to represent panting and were removed from analyses.

**Figure 1:**
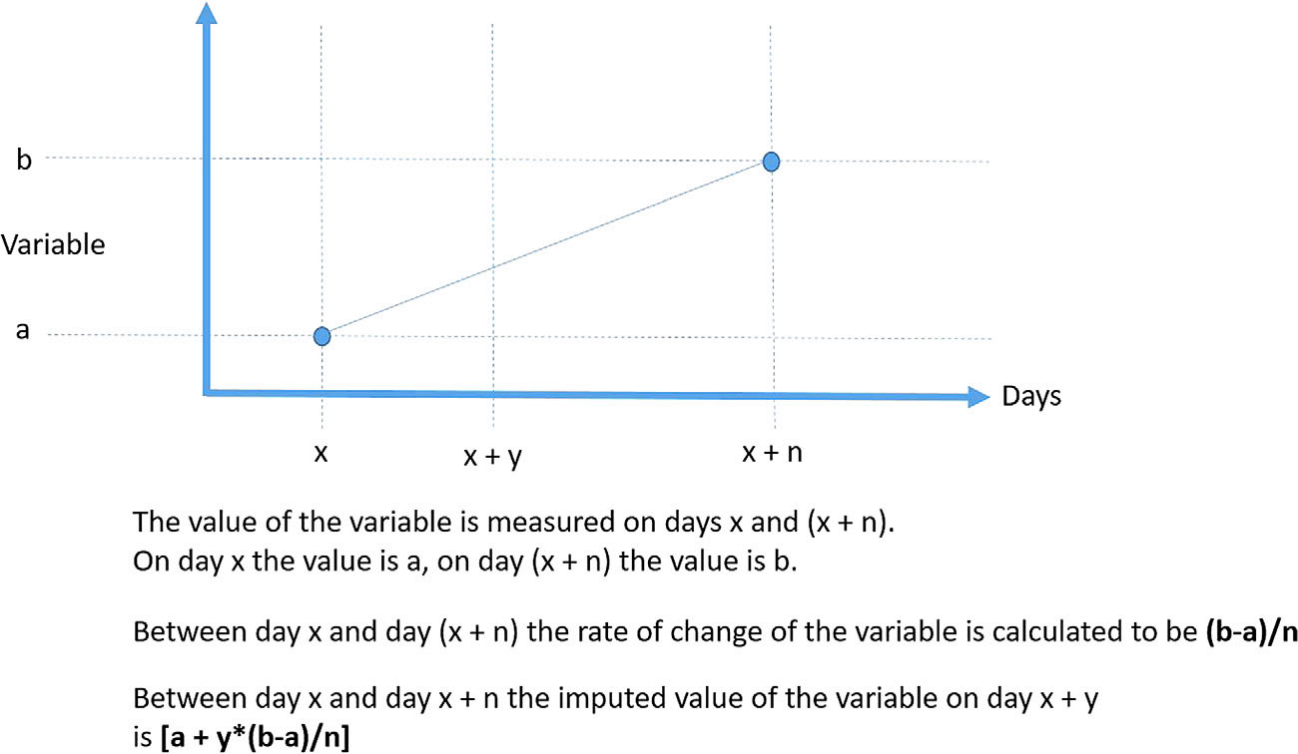
The method of calculation of imputed values of continuous variables and the rate of change of variables

For the 6 continuous variables recorded at least once in every 8‐month period throughout the study (HR, RR, RRR, RT, BW, and VHS) for which at least 2 measurements at different time points were available for any given dog, the following were calculated: An absolute value of the variable for each dog on any day between 2 observations was calculated by interpolation for the days on which the value was not known. Absolute values of variables between visits were calculated by plotting values obtained at each visit against time for each dog, assuming a linear rate of change, and interpolating values from the plotted line to give the value of that variable for each intervening day. The rate of change of the variable was calculated for each day a dog was in the study as illustrated in Figure 1. The rate of change was assumed to be constant between 2 observations.

The daily mean of the absolute values and the daily rate of change for each group (CHF and no‐CHF) were calculated for each day and plotted against time. Values for rate of change were expressed as the rate of change per month, calculated by multiplying the daily rate of change by 30. These were plotted initially by treatment group (pimobendan and placebo) and with data from dogs in both treatment groups pooled. Graphs display the curves for the 18 months (540 days) evaluated before day 0.

Finally, for the CHF group, to illustrate the temporal relationships between the different changes observed, the rates of change were expressed as a percentage of the maximum rate of change for each variable. Variables that increased with time were plotted on 1 set of axes. Variables that decreased with time were plotted on a different set of axes. No inferential statistics were performed.

## 3 RESULTS

### 3.1 Dogs

Over the duration of the study, 135 dogs experienced an onset of CHF and were included in the CHF group. Of the dogs in the CHF group, 59 were receiving pimobendan and 76 placebo.

The no‐CHF group consisted of 73 dogs. Of the dogs in the no‐CHF group, 50 were receiving pimobendan and 23 placebo. The median time in study for the CHF dogs was 414 days (interquartile range [IQR] 242‐729 days). The median time in study for the no‐CHF dogs was 1055 days (IQR, 976‐1221 days).

The baseline characteristics of the 2 groups are summarized in Table 2. Twenty‐eight from a total of 951 observations (2.9%) of RR were ≥100 breaths per minute and therefore were excluded from analyses. Twenty‐five of these excluded observations were made in 18 dogs in the CHF group (4.0% of all observations in this group). In only 4 instances were these excluded observations made at the time of onset of CHF. Three RR observations were excluded in 3 dogs in the no‐CHF group (0.9% of all observations in this group). In none of these instances were the excluded observations made at the final visit. Three from a total of 859 observations of RRR were ≥100 breaths per minute and therefore were excluded from analyses. All 3 observations were in the CHF group representing 0.6% of all observations in this group. All 3 observations corresponded to the visit at which CHF was diagnosed. One dog in the CHF group was excluded from analyses of VHS as consequence of there being a single obvious outlying data point where the magnitude of change over consecutive measurements was considered more likely to be attributable to a measurement or entry error rather than to genuine progression of disease.

**Table 2:** Baseline characteristics of the Group CHF and Group no‐CHF

	Variable	Group	
		CHF, N = 135	No‐CHF, N = 73
Dog characteristics	Proportion on pimobendan versus placebo (%)	Pimobendan = 59 (43.7%) Placebo = 76 (56.3%)	Pimobendan = 50 (68.5%) Placebo = 23 (31.5%)
	Age (years)	9.0 (7.0‐11.0)	9.0 (7.0‐10.0)
	Sex (M/F/MC/FS) (%)	17.8/4.4/43.0/34.8	26.0/5.5/35.6/32.9
	Breed (CKCS/Yorkshire terrier Dachshund/Poodle/Miniature Schnauzer/mixed breed/other breeds) (%)	45.9/2.2/5.9/3.0/3.0/11.1/28.9	45.2/1.4/4.1/2.7/5.5/6.8/34.2
Physical Examination	Body weight (kg)	9.0 (7.2‐10.7)	8.3 (6.3‐10.4)
	Resting respiratory rate (breaths per min if <100)	23 (19‐28) **[*N = 120]***	21 (18‐26) **[*[N = 71]***
	Respiratory rate (breaths per min if <100)	30 (24‐36) **[*N = 127*]**	28 (24‐36) **[*N = 67*]**
	Heart rate (BPM)	128 (111‐140)	120 (106‐140)
	Rectal temperature (°C)	38.7 (38.4‐39.1)	38.7 (38.4–39.1) **[*N = 71*]**
Diagnostic Imaging	Vertebral heart sum	11.4 (11.0‐12.0) **[*N = 134*]**	11.5 (11.0‐12.0)

*Note:* Continuous variables are reported as median (interquartile range). Categorical variables are reported as number (%). The number of observations is reported for variables where it is different to the reported number of dogs in the group.

### 3.2 Continuous variables and their rate of change

**Figure 2:**
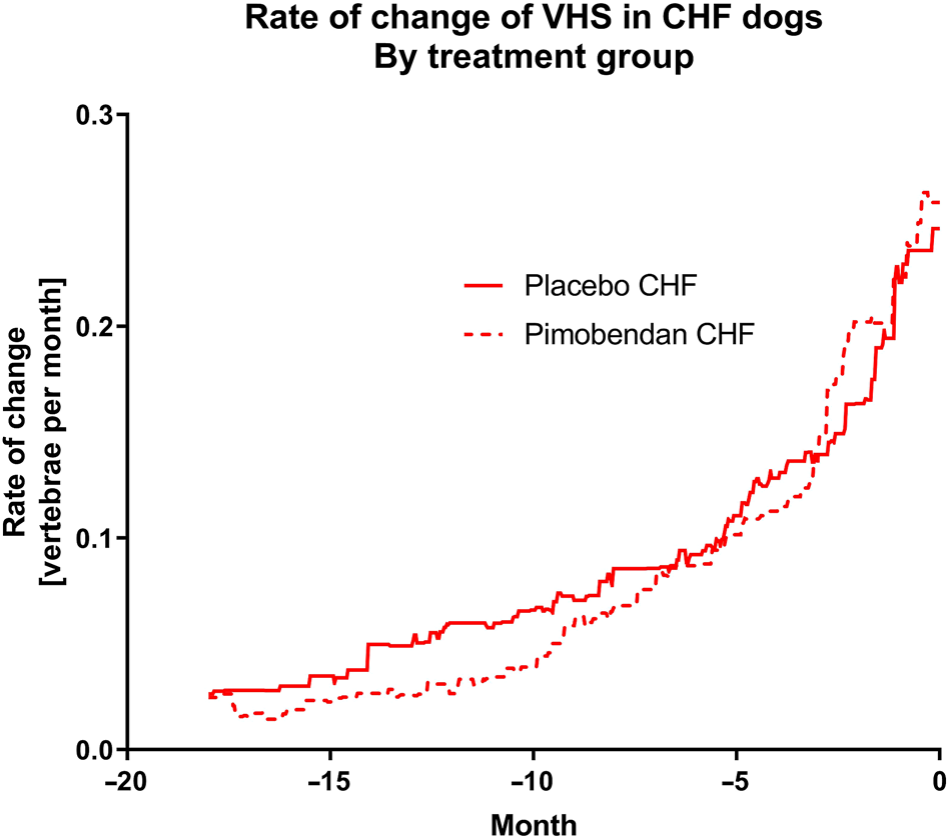
The rate of change of vertebral heart sum in dogs in the CHF group separated according to the treatment they were receiving

In all instances, visual inspection of plots showing absolute change and rate of change in the 6 studied variables in dogs receiving pimobendan or placebo suggested that, within the CHF group and the no‐CHF group, the pimobendan and placebo groups did not behave differently. This is illustrated for the rate of change of VHS in the CHF group in Figure 2 (similar graphs illustrating the rate of change for the CHF population in the 2 treatment groups are available in Figure S1A‐E). All other displayed graphs are derived from data pooling both treatment groups (pimobendan and placebo) within each group (group CHF and group no‐CHF).

**Figure 3:**
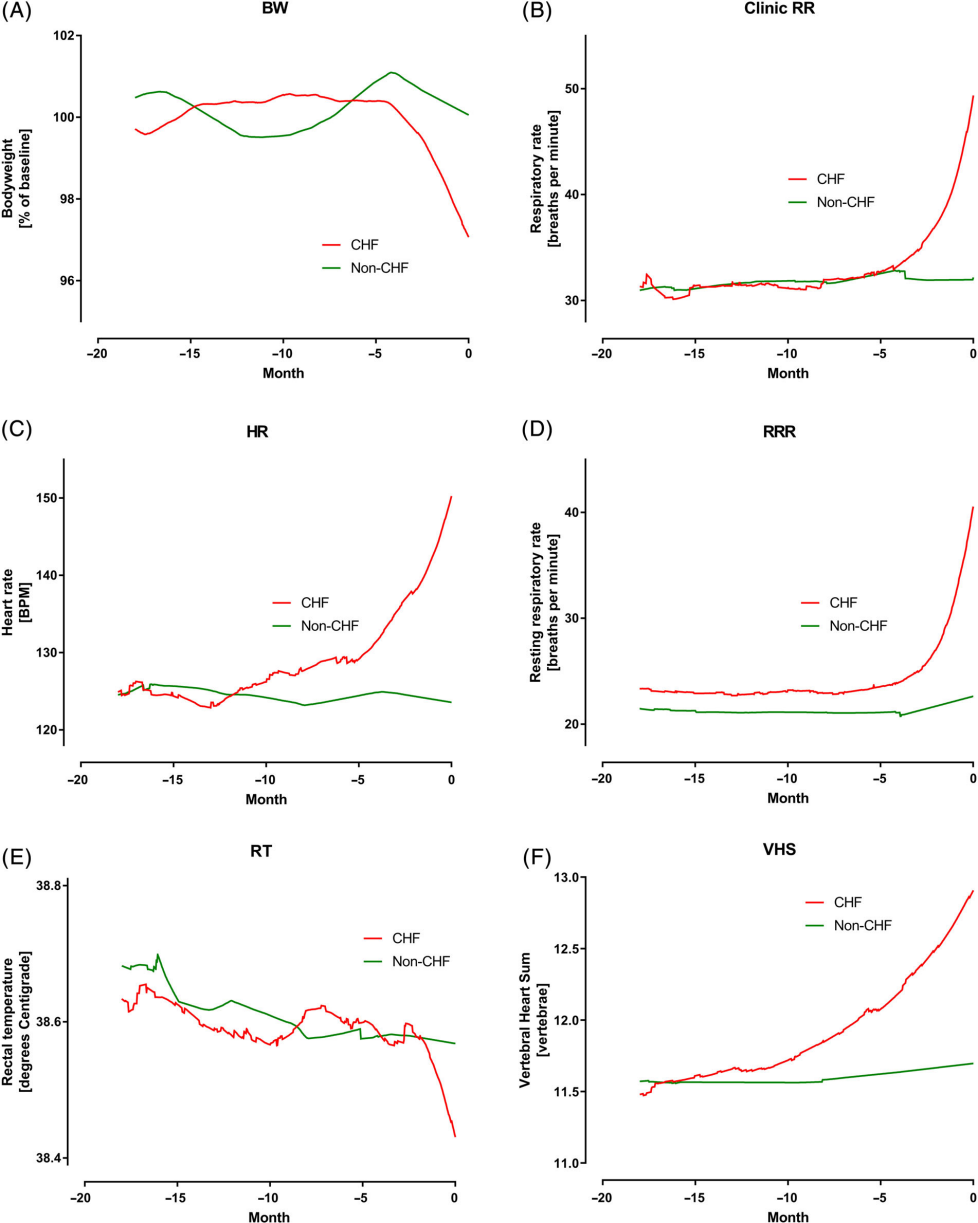
A, Percent of baseline bodyweight (BW) in the 2 groups over the 18 months before day 0. B, Clinic measured respiratory rate (RR) in the two groups over the 18 months before day 0. C, Heart rate (HR) in the 2 groups over the 18 months before day 0. D, Owner measured resting respiratory rate (RRR) in the 2 groups over the 18 months before day 0. E, Rectal temperature (RT) in the 2 groups over the 18 months before day 0. F, Vertebral heart sum (VHS) in the 2 groups over the 18 months before day 0

The absolute values of the 6 studied variables for the CHF and no‐CHF dogs are shown in Figure 3A‐F. Body weight remained at approximately 100% of baseline for both groups until approximately 4 months before day 0. In the CHF group, mean BW decreased by approximately 3.5% in the 4 months before day 0. No similar change was seen in the non‐CHF group. Mean RR remained similar at approximately 31 breaths per minute in both groups until 4 months before day 0. In the CHF group, the mean RR then increased to 49 breaths per minute by day 0 while remaining approximately 30 breaths per minute in the non‐CHF group. Mean RRR was in the low 20s in both groups until approximately 4 months before day 0. Over the last 4 months, mean RRR increased to approximately 40 breaths per minute in the CHF group while remaining constant in the non‐CHF group. Mean HR was approximately 125 bpm in both groups until about 10 months before day 0. Over the 10 months before day 0, it increased to approximately 150 bpm in the CHF group while remaining at approximately 125 in the non‐CHF group. Mean RT was approximately 38.6°C in both groups until the final 2 months before CHF. In the last 2 months, RT decreased by approximately 0.2°C in the CHF group while remaining 38.6°C in the non‐CHF group. Finally, mean VHS initially was approximately 11.5 in both groups. In the CHF group the mean VHS gradually increased over the 12 months before day 0 to 12.9 at the onset of CHF. In the non‐CHF group, VHS did not change over the 18‐month period.

**Figure 4:**
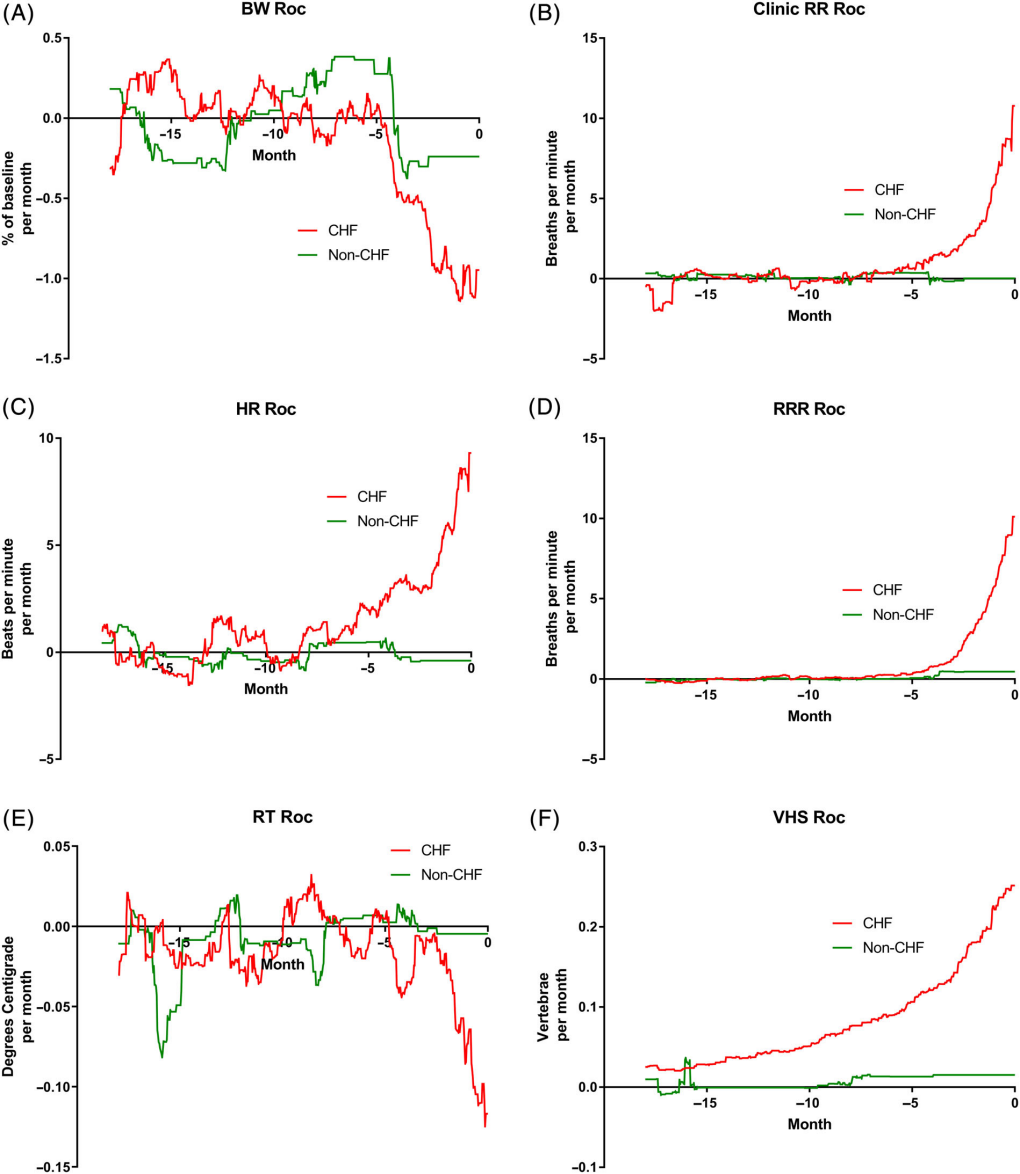
A, Rate of change (Roc) of bodyweight (BW) in the 2 groups over the 18 months before day 0. B, Roc of clinic measured respiratory rate (RR) in the 2 groups over the 18 months before day 0. C, Roc of heart rate (HR) in the 2 groups over the 18 months before day 0. D, Roc of owner measured resting respiratory rate (RRR) in the 2 groups over the 18 months before day 0. E, Roc of rectal temperature (RT) in the 2 groups over the 18 months before day 0. F, Roc of vertebral heart sum (VHS) in the 2 groups over the 18 months before day 0

**Figure 5:**
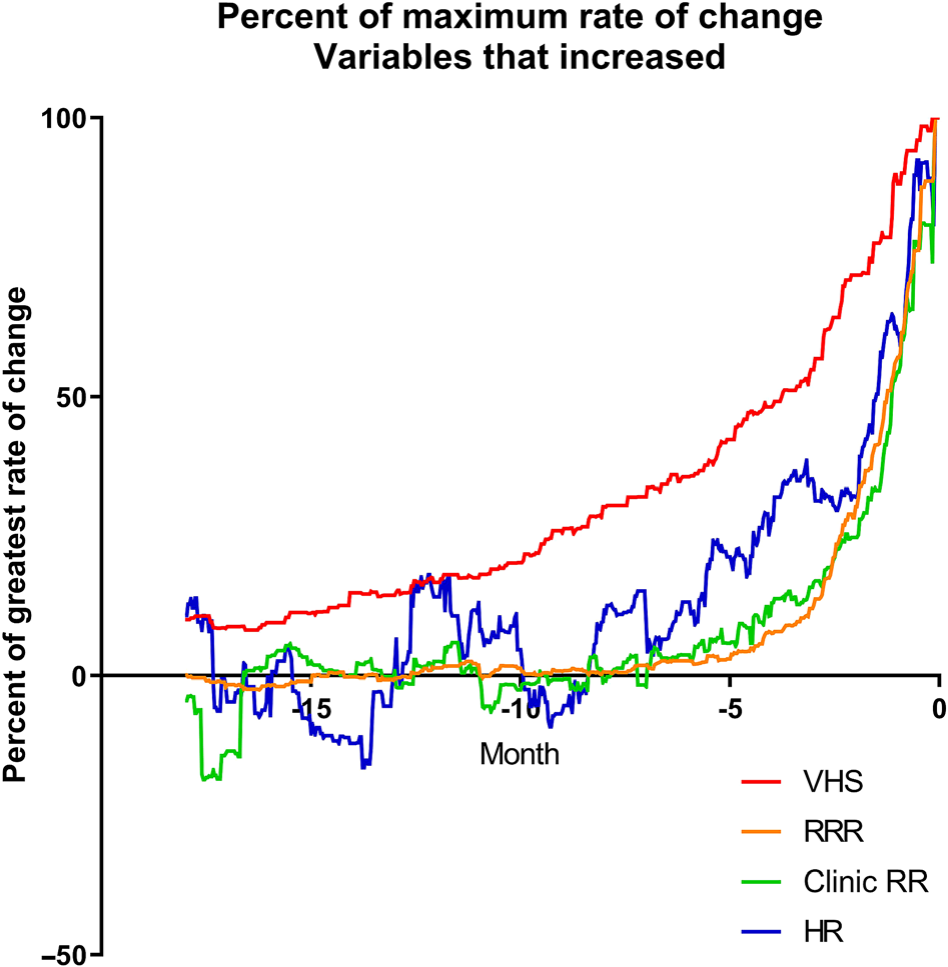
Percent of the maximum rate of change for the 4 variables that increased over time expressed against time in the 18 months up to and including day 0

**Figure 6:**
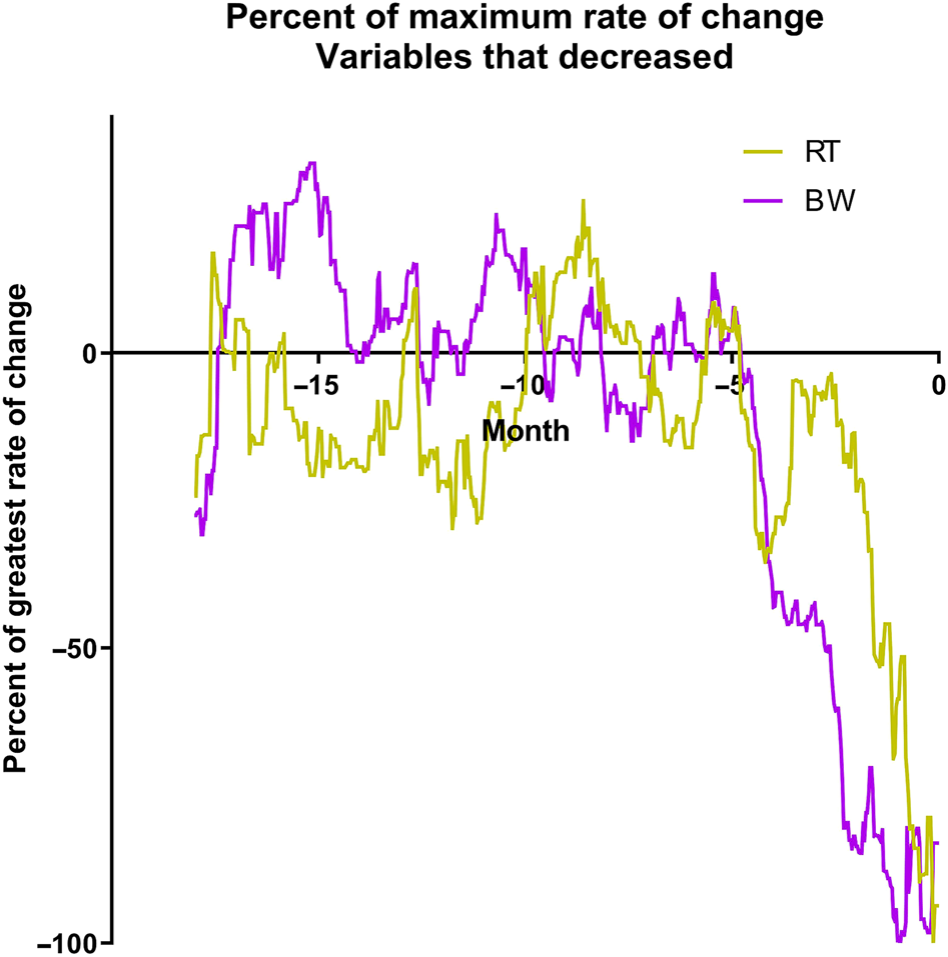
Percent of the maximum rate of change for the 2 variables that decreased over time expressed against time in the 18 months up to and including day 0

The rate of change per month of the 6 studied variables is shown in Figure 4A‐F. The temporal relationship of the maximum change of the 4 studied variables in the CHF dogs that increased is shown in Figure 5. The temporal relationship of the maximum change of the 2 variables in the CHF dogs that decreased is shown in Figure 6. For all variables, the maximal rate of change was observed in the period immediately before the onset of CHF.

## 4 DISCUSSION

Ours is the first study to demonstrate the time course over which changes in several clinical variables occur in dogs with MMVD as they progress into CHF. The changes that occurred in these variables were contrasted to those that occurred over a similar time period in dogs known not to have developed CHF.

Our study showed that all evaluated variables (HR, RR, RRR, RT, BW and VHS) changed in MMVD stage B2 dogs as they developed CHF. Similar changes were not observed in dogs that did not progress into CHF.

The most striking changes in any of the variables were found in RR and RRR. These increased from baseline values of approximately 31 and 23 per minute, respectively, to 49 and 41 per minute at the onset of CHF. These changes represent increases of 58 and 78% of the baseline value, respectively. The RRR values generally were lower than the RR, presumably as a consequence that RR were measured at the clinic and under more stressful circumstances. Previous studies have shown that RRR >30 breaths per minute in home environment^11^ or RR >40 breaths per minute in clinic environment^9^ are strong indicators for the presence of active CHF in MMVD dogs without any other important comorbidity. Our findings agree with these cutoffs and show that RR and RRR are comparably stable during the progression into CHF until late stages. Both RR and RRR only appeared to change within the 4 months immediately before the onset of CHF and showed little variation before that time. It is also interesting to note that a lower proportion of values for RRR were excluded from analyses because of presumed panting. This may suggest that measures of RRR are less likely to be confounded by panting and, being obtained by averages obtained over several days, are less prone to being affected by outlying observations.

A nonlinear increase in VHS previously has been described in dogs and the findings of the present study agree with these studies^3^, ^4^ (ie, the changes were characterized by a gradual increase over time until 6‐12 months before confirmed CHF when average VHS changed rapidly from approximately 11.5 to almost 13.0, representing an approximate increase of 15%). This increase in VHS represents an increase in heart size probably brought about by worsening volume overload and worsening of MR. The latter may be caused by worsening of the myxomatous changes in the valve leaflets, cardiac dilatation itself, and possibly chordal ruptures of the mitral valve.

Heart rate previously has been shown to increase over time in MMVD dogs as they progress into CHF.^6^, ^16^ In our study, small increases in HR could be seen from 10 months before confirmed CHF, but no such change could be observed in dogs not progressing to CHF within 4 months of the last examination. Mean heart rate increased from approximately 125 to 150 beats per minute, representing a change of 20% from the baseline value. The increase in HR probably is brought about by a response to worsening MR causing a decrease in forward stroke volume, which is compensated by increased sympathetic tone leading to increased HR.^17^, ^18^

Body weight and RT both decreased over time as dogs progressed into CHF. The decrease in RT is likely to be a consequence of impaired peripheral perfusion.^19^ The decrease in BW may be a consequence of the loss of skeletal muscle mass, but we did not record body condition or muscle condition scores at each visit and therefore cannot be sure of this conclusion. Loss of muscle mass may occur as a consequence of cardiac cachexia.^20^ Cardiac cachexia is a complex syndrome that may be, at least in part, a consequence of systemic inflammation^21^ and intestinal underperfusion.^22^ Furthermore, during the development of CHF, dogs may show evidence of a decreased appetite, which will decrease calorie intake and predispose to weight loss.^20^ Decreased appetite previously has been associated with worse outcome, both in the present study population of EPIC trial dogs^2^ and other studies.^23^ Cardiac cachexia has been associated with shorter survival times in dogs with heart disease.^21^, ^24^

The magnitude of the change observed in both BW and RT were comparably small in relation to the natural variability observed; an approximately 3% change in BW and 0.2°C change in RT. This observation indicates that, although these changes were evident on a population level, they are very unlikely to be detectable in an individual dog. A previous study reported a difference in RT of 0.3°C between dogs in stages B2 and C of MMVD, however the difference observed was not significant, perhaps because of the low number of dogs in the study.^19^ Although some definitions of cachexia require the loss of 5% of a dog's BW for it to be considered cachexic,^21^ our findings suggest that smaller decreases in BW are common in dogs developing CHF and not seen in dogs that do not develop CHF. It seems likely that similar mechanisms underlie the observed weight loss in these dogs.

The most important findings of our study illustrate the temporal evolution of the observed changes in the studied variables in MMVD stage B2 dogs before the onset of confirmed CHF. For all of the 6 variables evaluated, the rate of change of the variable was maximal in the period immediately before the onset of CHF (Figures 5 and 6). Although rate of change increased in VHS the closer in time the dogs were to CHF, the change was gradual over time compared to changes in the other variables and seemed to be evident at least 1 year before the onset of CHF. By contrast, the changes in BW, HR, RT, RR, and RRR occurred late in the process (ie, within the last few months before confirmed CHF). The magnitude and timing of the increase in respiratory rate variables reinforces the value of these variables as indicators of impending or incipient CHF.

### 4.1 Limitations

Not all dogs enrolled in the EPIC trial were included in the present study. The main group of interest consisted of only those dogs that progressed into verified CHF during the study. For comparison, we chose to characterize changes over a similar period in dogs that were known not to have developed CHF for at least 4 months after their final examination. The reason for the latter was to ensure that dogs in the comparison group could not have developed CHF shortly after their final examination. Dogs reaching the primary endpoint for reasons other than developing CHF were not included in the analysis. Dogs censored for reasons other than still being alive at the end of the study were not included because reasons for censoring these dogs could be associated with the possible development of CHF that was not verified as a primary endpoint (eg, because of administration of unauthorized drugs). Choosing to contrast the changes in the 2 extremes of the population (those that developed CHF and those that definitely did not) may exaggerate the observed differences and such clear contrasts may not be observed in a more heterogenous population.

In the present study, the maximum duration of time over which variables were described was 18 months. Many dogs, particularly those in the no‐CHF group, spent longer in the study, but the reason for selecting 18 months was that larger numbers of dogs could contribute observations over the entirety of this period leading to more precise estimates of variables.

Our study was limited to characterizing the changes in only 6 continuous variables. The reason for this limitation was that these were the only continuous variables measured at least once in every 8‐month period throughout the study. Accordingly, the study cannot characterize changes in other potential variables of interest, such as heart murmur intensity, echocardiographic findings, blood pressure, and biomarker concentrations.

The frequency of measured variables was limited to every 4 months for the clinical variables and every 8 months for VHS measurement. The changes occurring between these points of measurements therefore are unknown. Furthermore, not all intervals were standardized, because some visits at which variables were assessed were emergency visits rather than visits scheduled in the protocol.

We described the mean value and mean rate of change in the 6 variables we have evaluated. Within a population of dogs, variability will exist in the absolute values and their rates of change. We have not attempted to perform subanalyses or to identify populations that may have different rates of change such as those in which ruptured chordae tendinae may cause sudden development of CHF in the absence of a marked increase in heart size.

As shown in Figure 1, our method of imputing values of variables assumed a linear rate of change of those variables between true observations, but our analyses indicate that the rate of change was not linear. This situation may have resulted in an underestimate of the rate of change of variables at the end of periods between observations and an overestimate of the rate of change at the beginning of periods between observations. The overall effect on the absolute values of variables calculated may have been to conclude that values increased sooner than would have truly been observed had they been measured more frequently. This may mean that changes in the variables that were only seen to occur late in the disease process in reality occurred even later, and more rapidly, than are illustrated in our figures.

Finally, on any given day, the mean values of the variables illustrated are based on an average of both observed and imputed values. This makes it impossible to calculate appropriate confidence intervals for the mean values illustrated in our graphs because calculation of such would require independent real measurements, which was not the case in the present study, causing an underestimation of the interval sizes.

## 5 CONCLUSIONS

Dogs with MMVD in ACVIM stage B2^12^ experience increases in HR, RR, RRR, and VHS, and decreases in BW and RT as they progress into CHF. The change in VHS is characterized by a gradual increase over time, whereas the other variables change within the last few months before CHF. The variables with highest absolute change and rate of change were RR and RRR. This finding reinforces the value of RR and RRR as indicators of impending or incipient CHF. If RR and RRR are routinely and frequently measured in dogs with stage B2 MMVD, it may be possible for the onset of CHF to be detected earlier and therefore for it to be managed sooner and more effectively.

## CONFLICT OF INTEREST DECLARATION

Philip Watson and Christoph Schummer are employees of Boehringer Ingelheim Animal Health GmbH. All other authors have received funding from Boehringer Ingelheim Animal Health GmbH within the last 5 years for some or all of the following activities: research, travel, speaking fees, consultancy fees, and preparation of educational materials. This manuscript does not directly describe the actions or effects of drugs manufactured by Boehringer Ingelheim Animal Health GmbH.

## OFF‐LABEL ANTIMICROBIAL DECLARATION

Authors declare no off‐label use of antimicrobials.

## INSTITUTIONAL ANIMAL CARE AND USE COMMITTEE (IACUC) OR OTHER APPROVAL DECLARATION

The study was approved by an ethical review committee at each site where this was required.

## HUMAN ETHICS APPROVAL DECLARATION

Authors declare human ethics approval was not needed for this study.

## Supporting information


**Figure S1**A Rate change (Roc) of body weight (BW) in dogs developing CHF, sub‐divided into the two treatment groups (Pimobendan and Placebo) over the 18 months prior to day zero.
**Figure S1**B: Rate change (Roc) of clinic measured respiratory rate (RR) in dogs developing CHF, sub‐divided into the two treatment groups (Pimobendan and Placebo) over the 18 months prior to day zero.
**Figure S1**C: Rate change (Roc) of heart rate (HR) in dogs developing CHF, sub‐divided into the two treatment groups (Pimobendan and Placebo) over the 18 months prior to day zero.
**Figure S1**D: Rate change (Roc) of home‐measured resting respiratory rate (RRR) in dogs developing CHF, sub‐divided into the two treatment groups (Pimobendan and Placebo) over the 18 months prior to day zero.
**Figure S1**E: Rate change (Roc) of rectal temperature (RT) in dogs developing CHF, sub‐divided into the two treatment groups (Pimobendan and Placebo) over the 18 months prior to day zero.
