# Supplementary figures and images for: Temporal changes in clinical and radiographic variables in dogs with preclinical myxomatous mitral valve disease: The EPIC study
Source: J Vet Intern Med. 2020 Mar 22;34(3):1108–18. doi: 10.1111/jvim.15753 (PMC7255670; doi:10.1111/jvim.15753)

## BW ROC two groups

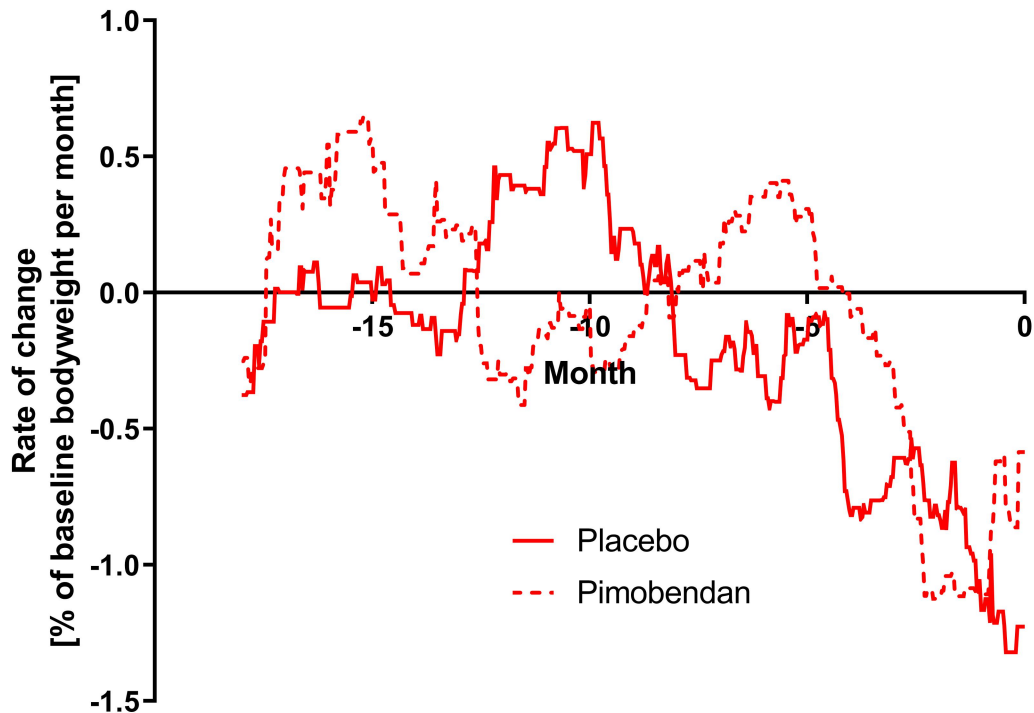

Supplement: Supplementary file 1 — Figure S1A Rate change (Roc) of body weight (BW) in dogs developing CHF, sub‐divided into the two treatment groups (Pimobendan and Placebo) over the 18 months prior to day zero. Figure S1B: Rate change (Roc) of clinic measured respiratory rate (RR) in dogs developing CHF, sub‐divided into the two treatment groups (Pimobendan and Placebo) over the 18 months prior to day zero. Figure S1C: Rate change (Roc) of heart rate (HR) in dogs developing CHF, sub‐divided into the two treatment groups (Pimobendan and Placebo) over the 18 months prior to day zero. Figure S1D: Rate change (Roc) of home‐measured resting respiratory rate (RRR) in dogs developing CHF, sub‐divided into the two treatment groups (Pimobendan and Placebo) over the 18 months prior to day zero. Figure S1E: Rate change (Roc) of rectal temperature (RT) in dogs developing CHF, sub‐divided into the two treatment groups (Pimobendan and Placebo) over the 18 months prior to day zero. [file JVIM-34-1108-s001.zip › JVIM_15753_Fig_S1A.pdf]

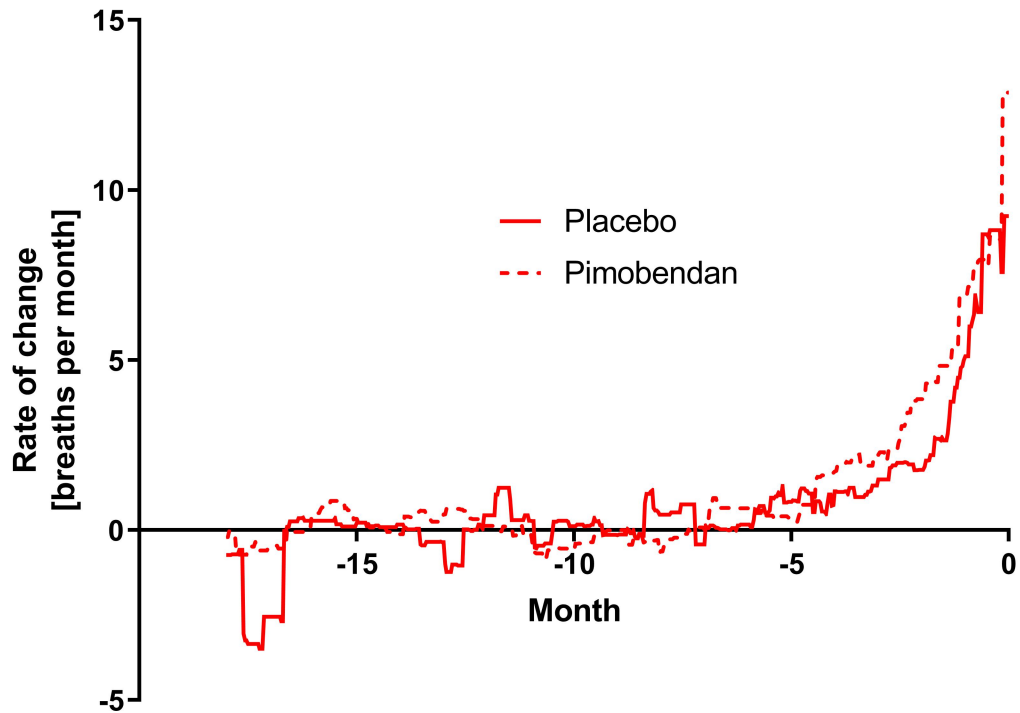

Supplement: Supplementary file 1 — Figure S1A Rate change (Roc) of body weight (BW) in dogs developing CHF, sub‐divided into the two treatment groups (Pimobendan and Placebo) over the 18 months prior to day zero. Figure S1B: Rate change (Roc) of clinic measured respiratory rate (RR) in dogs developing CHF, sub‐divided into the two treatment groups (Pimobendan and Placebo) over the 18 months prior to day zero. Figure S1C: Rate change (Roc) of heart rate (HR) in dogs developing CHF, sub‐divided into the two treatment groups (Pimobendan and Placebo) over the 18 months prior to day zero. Figure S1D: Rate change (Roc) of home‐measured resting respiratory rate (RRR) in dogs developing CHF, sub‐divided into the two treatment groups (Pimobendan and Placebo) over the 18 months prior to day zero. Figure S1E: Rate change (Roc) of rectal temperature (RT) in dogs developing CHF, sub‐divided into the two treatment groups (Pimobendan and Placebo) over the 18 months prior to day zero. [file JVIM-34-1108-s001.zip › JVIM_15753_Fig_S1B.pdf]

## HR ROC two groups

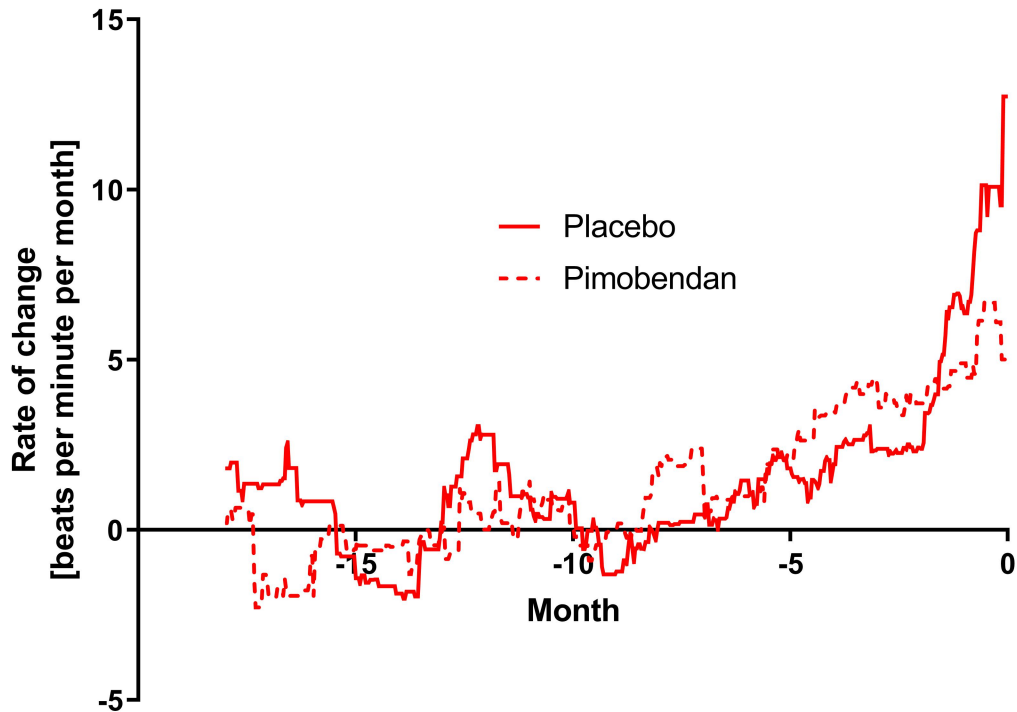

Supplement: Supplementary file 1 — Figure S1A Rate change (Roc) of body weight (BW) in dogs developing CHF, sub‐divided into the two treatment groups (Pimobendan and Placebo) over the 18 months prior to day zero. Figure S1B: Rate change (Roc) of clinic measured respiratory rate (RR) in dogs developing CHF, sub‐divided into the two treatment groups (Pimobendan and Placebo) over the 18 months prior to day zero. Figure S1C: Rate change (Roc) of heart rate (HR) in dogs developing CHF, sub‐divided into the two treatment groups (Pimobendan and Placebo) over the 18 months prior to day zero. Figure S1D: Rate change (Roc) of home‐measured resting respiratory rate (RRR) in dogs developing CHF, sub‐divided into the two treatment groups (Pimobendan and Placebo) over the 18 months prior to day zero. Figure S1E: Rate change (Roc) of rectal temperature (RT) in dogs developing CHF, sub‐divided into the two treatment groups (Pimobendan and Placebo) over the 18 months prior to day zero. [file JVIM-34-1108-s001.zip › JVIM_15753_Fig_S1C.pdf]

## RRR ROC two groups

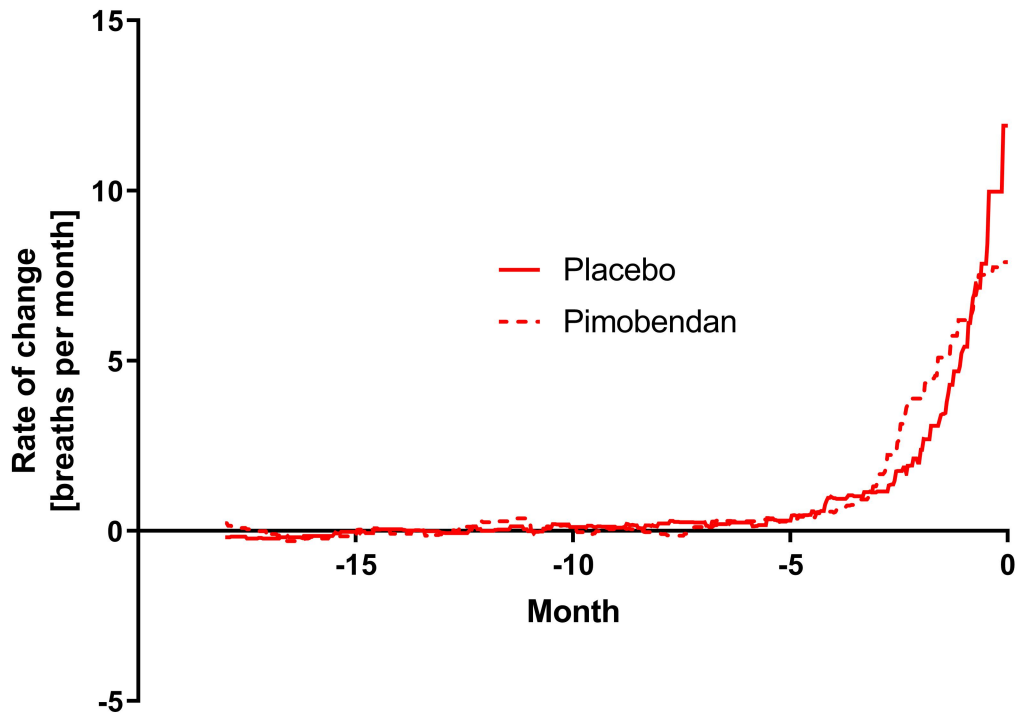

Supplement: Supplementary file 1 — Figure S1A Rate change (Roc) of body weight (BW) in dogs developing CHF, sub‐divided into the two treatment groups (Pimobendan and Placebo) over the 18 months prior to day zero. Figure S1B: Rate change (Roc) of clinic measured respiratory rate (RR) in dogs developing CHF, sub‐divided into the two treatment groups (Pimobendan and Placebo) over the 18 months prior to day zero. Figure S1C: Rate change (Roc) of heart rate (HR) in dogs developing CHF, sub‐divided into the two treatment groups (Pimobendan and Placebo) over the 18 months prior to day zero. Figure S1D: Rate change (Roc) of home‐measured resting respiratory rate (RRR) in dogs developing CHF, sub‐divided into the two treatment groups (Pimobendan and Placebo) over the 18 months prior to day zero. Figure S1E: Rate change (Roc) of rectal temperature (RT) in dogs developing CHF, sub‐divided into the two treatment groups (Pimobendan and Placebo) over the 18 months prior to day zero. [file JVIM-34-1108-s001.zip › JVIM_15753_Fig_S1D.pdf]

## RT ROC two groups

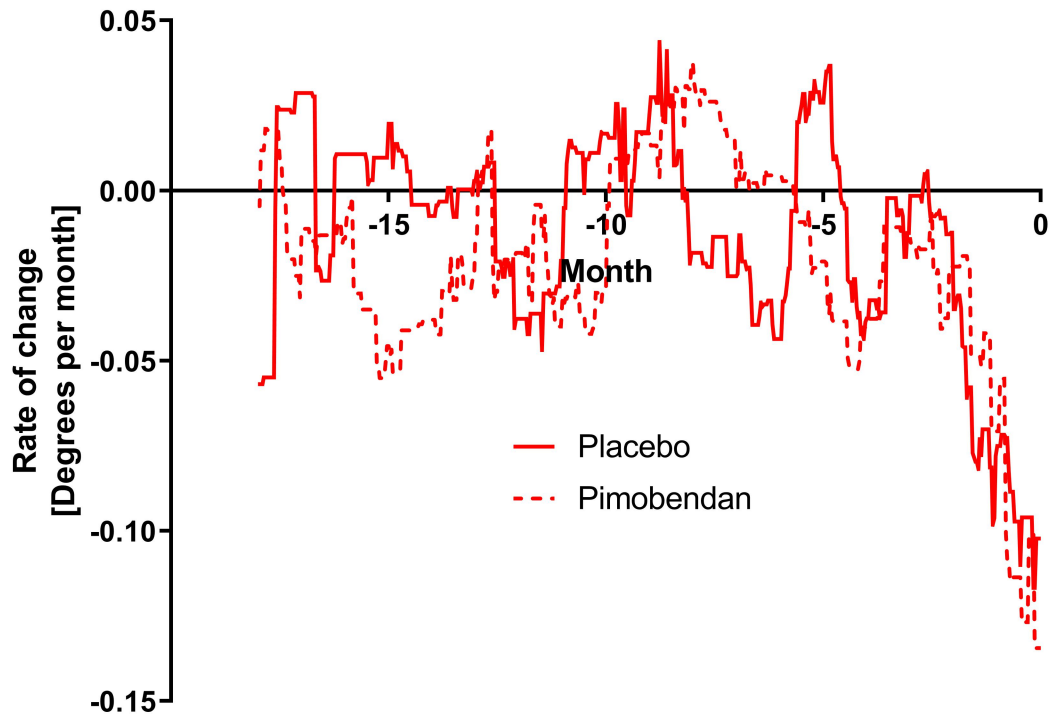

Supplement: Supplementary file 1 — Figure S1A Rate change (Roc) of body weight (BW) in dogs developing CHF, sub‐divided into the two treatment groups (Pimobendan and Placebo) over the 18 months prior to day zero. Figure S1B: Rate change (Roc) of clinic measured respiratory rate (RR) in dogs developing CHF, sub‐divided into the two treatment groups (Pimobendan and Placebo) over the 18 months prior to day zero. Figure S1C: Rate change (Roc) of heart rate (HR) in dogs developing CHF, sub‐divided into the two treatment groups (Pimobendan and Placebo) over the 18 months prior to day zero. Figure S1D: Rate change (Roc) of home‐measured resting respiratory rate (RRR) in dogs developing CHF, sub‐divided into the two treatment groups (Pimobendan and Placebo) over the 18 months prior to day zero. Figure S1E: Rate change (Roc) of rectal temperature (RT) in dogs developing CHF, sub‐divided into the two treatment groups (Pimobendan and Placebo) over the 18 months prior to day zero. [file JVIM-34-1108-s001.zip › JVIM_15753_Fig_S1E.pdf]
